# Physiological Responses to a Sequential Higher-Frequency Low Tidal Volume Ventilation Protocol During Radiofrequency Atrial Fibrillation Ablation: A Single-Center Subgroup Proof-of-Concept Study

**DOI:** 10.3390/life16101654

**Published:** 2026-10-01

**Authors:** Max Briefs, Maximilian A. Jacobi, Astrid Bergmann, Marc P. Woldt, Philipp Sommer, Vera von Dossow

**Affiliations:** 1 Heart and Diabetes Center NRW, Institute of Anesthesiology and Pain Therapy, Ruhr University Bochum, 32545 Bad Oeynhausen, Germany; maximilian.jacobi@ruhr-uni-bochum.de (M.A.J.); asbergmann@hdz-nrw.de (A.B.); mpwoldt@hdz-nrw.de (M.P.W.); vvondossow@hdz-nrw.de (V.v.D.); 2 Heart and Diabetes Center NRW, Department of Electrophysiology, Ruhr University Bochum, 32545 Bad Oeynhausen, Germany; psommer@hdz-nrw.de

**Keywords:** mechanical ventilation, higher-frequency ventilation, radiofrequency ablation, proof of concept study

## Abstract

The physiological effects of mechanical ventilation are subject to ongoing research. Conventional ventilation may contribute to pulmonary volutrauma or barotrauma and influence cardiovascular physiology through changes in intrathoracic pressure. In the multicenter Ablate-by-LAWT study, patients undergoing atrial fibrillation ablation were ventilated using a higher-frequency, low tidal ventilation (HFLTV) protocol. This post hoc single-center subgroup proof-of-concept study aimed to characterize physiological responses to HFLTV using left atrial pressure (LAP), gas exchange, and hemodynamic parameters during sequential, nonrandomized volume-controlled ventilation (VCV) and HFLTV. The ventilation protocols differed in respiratory rate, tidal volume, PEEP, FiO_2_, and I:E ratio. Twelve patients were included, and differences between ventilation strategies were assessed using two-sided paired *t*-tests. Compared with VCV, HFLTV was associated with lower systolic LAP (Mean Difference (MD) −3.33 mmHg, 95% confidence interval (CI) [−5.61; −1.06], *p* = 0.008), whereas post hoc estimated mean LAP was similar between ventilation periods (MD −0.06 mmHg, 95% CI [−2.19; 2.08], *p* = 0.955). Heart rate (MD 8.1 bpm, 95% CI [3.8; 12.4], *p* = 0.002), mean arterial pressure (MD 11.5 mmHg, 95% CI [7.1; 15.9], *p* < 0.001), and cardiac index (MD 0.64 L/min/m^2^, 95% CI [0.29; 0.99], *p* = 0.002) were higher during HFLTV. The partial arterial pressure of oxygen (P_a_O_2_) was lower during HFLTV (MD −42.3 mmHg, 95% CI [−83.5; −1.1], *p* = 0.045, FDR-adjusted *p* = 0.072), and the partial arterial pressure of carbon dioxide (P_a_CO_2_) (MD −1.1 mmHg, 95% CI [−6.0; 3.7], *p* = 0.623) and the P_a_O_2_/FiO_2_ ratio (MD −49.3 mmHg, 95% CI [−105.2; 6.7], *p* = 0.079) did not differ significantly. Given the fixed-order design and simultaneous modification of multiple ventilatory parameters, these exploratory findings should be considered hypothesis-generating and cannot be attributed to individual components of the HFLTV protocol.

## 1. Introduction

Mechanical ventilation is an essential component of general anesthesia and is associated with changes in intrathoracic pressure that may influence both pulmonary and cardiovascular physiology [1]. Conventional volume-controlled ventilation (VCV) commonly applies moderate tidal volumes to ensure adequate gas exchange; however, mechanical stress resulting from excessive alveolar distension or pressure load may contribute to ventilation-associated lung injury [2]. In addition, positive-pressure ventilation affects venous return, ventricular loading conditions, and cardiac function through alterations in intrathoracic pressure [3].

Lung-protective ventilation strategies have therefore increasingly focused on reducing tidal volume to limit mechanical stress on the respiratory system [4]. Lower tidal volumes have been associated with reduced inflammatory response and fewer postoperative pulmonary complications, with the reduction in tidal volume being considered a major contributor to these effects [5,6,7]. Further reduction in tidal volume requires compensatory adjustments, such as increased respiratory frequency, to maintain adequate minute ventilation, which has prompted the investigation of higher-frequency ventilation [8].

The multicenter Ablate-by-LAWT study investigated a personalized ablation strategy for paroxysmal atrial fibrillation (AF) based on local left atrial wall thickness (LAWT) [9]. According to the study protocol, patients underwent general anesthesia with a standardized sequential higher-frequency, low tidal ventilation (HFLTV) protocol. This provided an opportunity to investigate physiological responses to HFLTV in a clinical procedural setting. In a subgroup of patients treated at one participating center, invasive left atrial pressure (LAP) measurements were available in addition to routinely recorded respiratory and hemodynamic parameters.

Higher-frequency low tidal ventilation combines increased respiratory frequency with reduced tidal volumes and aims to decrease respiratory excursion while maintaining effective gas exchange. This approach may be particularly relevant during interventional procedures in which respiratory motion can affect procedural accuracy, such as catheter-based electrophysiological interventions. A previous single-center subgroup analysis of the Ablate-by-LAWT study investigated the effects of high-frequency, very low tidal volume ventilation with controlled peak inspiratory pressure limitation (HFvLTV-cPP) during AF ablation [10]. HFvLTV-cPP was associated with a reduction in respiration-related catheter displacement, permissive hypercapnia and preserved oxygenation, with hemodynamic parameters remaining within predefined safety limits [10]. The authors interpreted these findings as evidence of short-term physiological feasibility rather than definitive evidence of clinical safety or benefit.

Although these findings support the feasibility of higher-frequency low tidal ventilation during AF ablation, further characterization of its physiological effects remains limited. In particular, invasive assessment of cardiac filling pressure and concurrent evaluation of respiratory and hemodynamic parameters may provide additional insight into the interactions between ventilation strategy and cardiovascular physiology. Such information may be relevant for future investigations in selected patient populations, including individuals with heart failure, pulmonary hypertension, or right ventricular dysfunction, in whom ventilation-related changes in cardiopulmonary interactions may be of particular interest.

The present study represents a single-center subgroup proof-of-concept analysis of twelve patients enrolled in the Ablate-by-LAWT study. The aim was to characterize physiological responses to HFLTV compared with conventional VCV by assessing changes in left atrial pressure (LAP), gas exchange, and selected hemodynamic parameters during catheter-based radiofrequency ablation.

## 2. Materials and Methods

**Figure 1:**
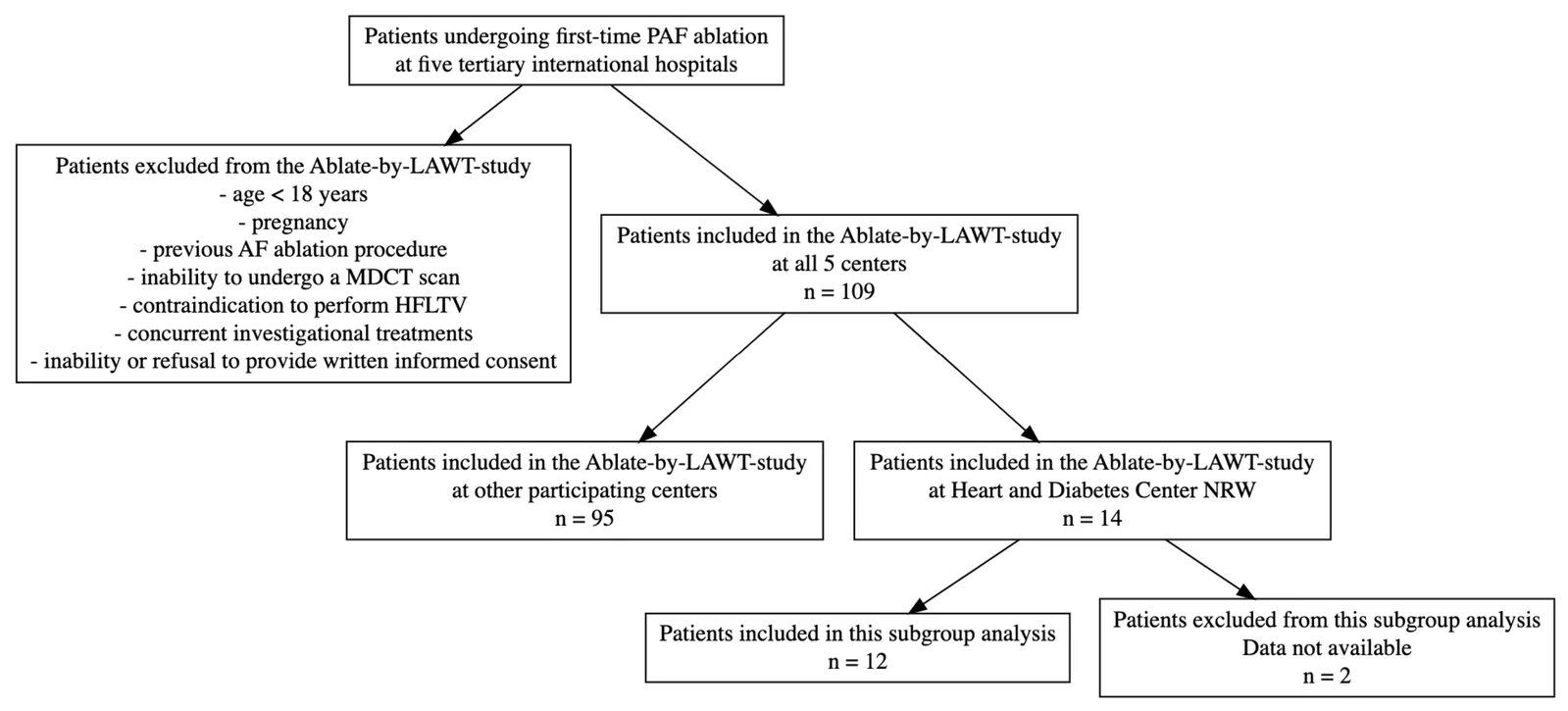
Flowchart of patient selection in the Ablate-by-LAWT study and this subgroup analysis. PAF: paroxysmal atrial fibrillation, LAWT: left atrial wall thickness, MDCT: multidetector computed tomography, HFLTV: higher-frequency low tidal volume ventilation.

This study represents a single-center subgroup proof-of-concept analysis of patients enrolled in the multicenter Ablate-by-LAWT study. All patients received an informed consent form regarding study participation and provided written informed consent prior to enrollment. The present analysis was conducted post hoc and was not prespecified in the Ablate-by-LAWT study protocol. No study-specific sample-size calculation was performed for this exploratory subgroup proof-of-concept analysis. The sample size was determined by the number of patients enrolled in the parent Ablate-by-LAWT study at the participating center for whom the study-specific HFLTV protocol and paired invasive LAP measurements during HFLTV and VCV were available. Detailed information on patient selection and exclusion criteria in the Ablate-by-LAWT study is displayed in Figure 1. The present analysis represents a convenience subgroup of the parent study rather than a prospectively powered cohort. A previously published pilot analysis included 52 patients enrolled in the Ablate-by-LAWT study at a different participating center [10]. None of the patients included in the present analysis were included in the previous publication. Thus, there was no patient-level overlap between the two cohorts, and all patient-level data and outcomes reported in the present analysis are derived from a distinct cohort and were not included in the previous publication.

All patients underwent general anesthesia according to the Ablate-by-LAWT study protocol. General anesthesia was induced with sufentanil 0.3–0.5 µg/kg, propofol 1–2 mg/kg, and rocuronium 0.6 mg/kg. Dexamethasone 4 mg was administered at induction. Anesthesia was maintained with continuous propofol (4–6 mg/kg/h) and remifentanil (0.15–0.20 µg/kg/min) infusions. Hemodynamic support was provided as clinically required using intermittent cafedrine/theodrenaline and, if additional vasopressor support was required, continuous norepinephrine infusion. Mechanical ventilation was initially performed using conventional VCV with a fraction of inspired oxygen (FiO_2_) of 40%, a tidal volume of 6–8 mL/kg and a respiratory rate of 12–15 breaths/minute. Following a 10 min recruitment phase, ventilation was sequentially changed to a higher-frequency, low tidal ventilation HFLTV protocol according to the Ablate-by-LAWT study protocol. The HFLTV protocol included a FiO_2_ of 50%, a tidal volume of 3.5 to 4 mL/kg, a respiratory rate of 45–55 breaths/minute, an inspiration-to-expiration ratio (I:E) of 1:1.5 and a positive end-expiratory pressure (PEEP) of 0 cmH_2_O. Thus, HFLTV is a bundled ventilation protocol comprising simultaneous changes in respiratory rate, tidal volume, PEEP, FiO_2_, and the I:E ratio, rather than an isolated modification of respiratory frequency or tidal volume. Total PEEP was not assessed using an end-expiratory occlusion maneuver, and consequently, intrinsic PEEP and dynamic hyperinflation could not be quantified. The reported PEEP values therefore represent the externally set PEEP and should not be interpreted as total end-expiratory pressure. Measured or applied ventilatory parameters during VCV and HFLTV are displayed in Table 1.

HFLTV was delivered using the standard anesthesia Flow-i workstation (Getinge AB, Göteborg, Sweden), which was routinely used for mechanical ventilation throughout the procedure and did not require a dedicated high-frequency ventilation device. HFLTV was maintained for the duration of the ablation procedure according to the study protocol. Prior to emergence from anesthesia, a second recruitment using VCV was performed and patients were subsequently extubated when relaxometry was sufficient. After extubation, patients were admitted to the electrophysiological recovery room.

Physiological measurements followed the fixed procedural sequence of the Ablate-by-LAWT protocol. Before induction of general anesthesia, baseline arterial blood gas measurements were obtained. Following induction, intubation, and an initial recruitment maneuver, patients were ventilated using VCV. Arterial blood gases and hemodynamic parameters, including heart rate (HR), mean arterial pressure (MAP), and cardiac index (CI), were recorded during this initial VCV period. HFLTV was subsequently initiated and maintained throughout the ablation procedure. Arterial blood gases and hemodynamic parameters were recorded 5 min after initiating HFLTV and then at 30 min intervals. After completion of the HFLTV period, ventilation was returned to VCV, and a second recruitment maneuver was performed before emergence from anesthesia. Further measurements were obtained 5 min after discontinuation of HFLTV, after extubation (EXT), and 1 h after admission to the recovery room (REC).

For the paired statistical comparison of VCV and HFLTV, the VCV value for HR, MAP, CI, partial arterial pressure of oxygen (P_a_O_2_), and carbon dioxide (P_a_CO_2_) was the measurement obtained during the initial VCV period after induction, intubation, and recruitment. Because several measurements were obtained during HFLTV, these measurements were averaged within each patient to obtain one patient-level HFLTV value for each parameter. Measurements obtained after discontinuation of HFLTV, after extubation, and in the recovery room were used to illustrate the chronological course of the physiological parameters but were not included in the paired VCV-versus-HFLTV comparison.

LAP followed a separate measurement schedule. A single invasive LAP measurement was obtained after initiating HFLTV and before initiating radiofrequency ablation, and a second was obtained later during VCV before extubation. Accordingly, the paired LAP analysis compared these two single measurements obtained at different procedural stages. Left atrial access was established by transseptal puncture as part of the standard workflow for catheter-based radiofrequency ablation. In response to the observed opposite changes in systolic and diastolic LAP, an estimated mean LAP was additionally derived post hoc as (systolic LAP + 2 × diastolic LAP)/3, and the systolic-to-diastolic LAP amplitude was post hoc calculated as systolic minus diastolic LAP. These derived measures were compared between ventilation conditions using paired analyses. Because mean LAP was not directly recorded from the atrial pressure waveform, the calculated value was considered a post hoc estimate rather than a directly measured mean LAP.

The measurement sequence was fixed and was neither randomized nor counterbalanced. Thus, measurements under HFLTV and VCV were obtained at different stages of the procedure rather than under temporally matched conditions.

Blood gas measurements were conducted using the ABL800 FLEX analyzer (Radiometer GmbH, Krefeld, Germany). Simultaneously, hemodynamic parameters including heart rate (HR), mean arterial pressure and cardiac index were recorded. Hemodynamic status was monitored during the procedure using an arterial line, which was connected to both a HemoSphere monitor (Edwards Lifesciences, Irvine, CA, USA) and a Philips IntelliVue (Philips Healthcare, Hamburg, Germany) anesthesia monitor. Cardiac output was continuously estimated from the arterial pressure waveform using a FloTrac sensor connected to the HemoSphere advanced monitoring platform (Edwards Lifesciences, Irvine, CA, USA). The FloTrac algorithm derives cardiac output from arterial waveform characteristics and patient-specific demographic variables and does not require external calibration. Cardiac index was calculated by indexing cardiac output to body surface area.

Statistical analyses and visualization were performed using R version 4.4.1 [11]. Baseline characteristics and procedural parameters were summarized descriptively.

For HR, MAP, CI, P_a_O_2_, and P_a_CO_2_, paired comparisons were performed between the measurements obtained during the initial VCV period after induction, intubation, and recruitment and the patient-level mean of all measurements obtained during HFLTV. Because FiO_2_ differed between the ventilation protocols (0.40 during VCV and 0.50 during HFLTV), the P_a_O_2_/FiO_2_ (P/F) ratio was additionally calculated as an FiO_2_-normalized measure of oxygenation. P/F ratios were calculated at the same patient-level VCV and HFLTV measurement conditions used for the comparison of arterial blood gases. The P/F ratio was analyzed using a two-sided paired *t*-test. Because PEEP also differed substantially between ventilation conditions, the P/F ratio was considered an exploratory measure of oxygenation rather than a measure isolating the effect of HFLTV. To address the asymmetry between the single initial VCV measurement and the patient-level mean of repeated HFLTV measurements, an additional sensitivity analysis compared the initial VCV measurement with the single measurement obtained 5 min after initiation of HFLTV using paired *t*-tests.

For LAP, paired comparisons were based on the single measurement obtained during HFLTV and the subsequent single measurement obtained during VCV before extubation. Thus, each paired *t*-test included one VCV and one HFLTV value per patient. Unadjusted two-sided *p*-values are reported for the individual comparisons. To account for multiplicity, *p*-values across the physiological comparisons were additionally adjusted using the Benjamini–Hochberg false discovery rate (FDR) procedure as a sensitivity analysis. Both unadjusted and FDR-adjusted *p*-values are reported. The analyses of post hoc estimated mean LAP and systolic-to-diastolic LAP amplitude were performed post hoc and were not included in the FDR sensitivity analysis of the original physiological outcomes.

No prespecified primary physiological endpoint was defined for this exploratory subgroup proof-of-concept analysis; therefore, all investigated hemodynamic and respiratory outcomes were considered exploratory.

Peripheral Oxygen Saturation (SpO_2_), arterial pH, and airway pressure measurements obtained during HFLTV were therefore additionally evaluated descriptively as short-term physiological observations. For SpO_2_ and arterial pH, repeated measurements obtained during HFLTV were averaged within each patient, and these patient-level means, which were summarized across the cohort. The ranges of all recorded HFLTV measurements are also reported. Vasoactive medication administered during HFLTV and the requirement for rescue ventilation, interruption of HFLTV, recruitment maneuvers for desaturation, or treatment of arrhythmia were assessed descriptively. Plateau pressure was not systematically recorded; consequently, driving pressure could not be reliably calculated.

The distribution of within-patient differences between ventilation conditions was assessed for each paired comparison using the Shapiro–Wilk test and visual inspection of Q–Q plots. Given the small sample size, individual patient-level paired observations were additionally visualized to assess the consistency of within-patient changes and potentially influential observations. As a non-parametric sensitivity analysis, all paired comparisons were additionally evaluated using the Wilcoxon signed-rank test. The Wilcoxon analyses were considered complementary to the paired *t*-test analyses. A significance level of *p* < 0.05 was applied for all statistical tests. Due to the exploratory proof-of-concept design and limited sample size, analyses were considered hypothesis-generating rather than confirmatory.

## 3. Results

A total of twelve patients were included in this single-center subgroup analysis. The mean age was 61.83 years, and five patients (41.67%) were female. The mean duration of anesthesia was 134.67 min, the mean duration of the intervention was 97.00 min, and the mean duration of HFLTV exposure was 85.67 min. Baseline demographic characteristics and procedural parameters are summarized in Table 2.

The ventilatory and respiratory parameters measured during VCV and HFLTV are summarized in Table 3. As expected from the protocol, HFLTV was characterized by a substantially higher respiratory rate and lower tidal volume, with a higher minute ventilation. Plateau pressure and total PEEP were not systematically measured during the study protocol; consequently, driving pressure and intrinsic PEEP could not be determined.

**Figure 2:**
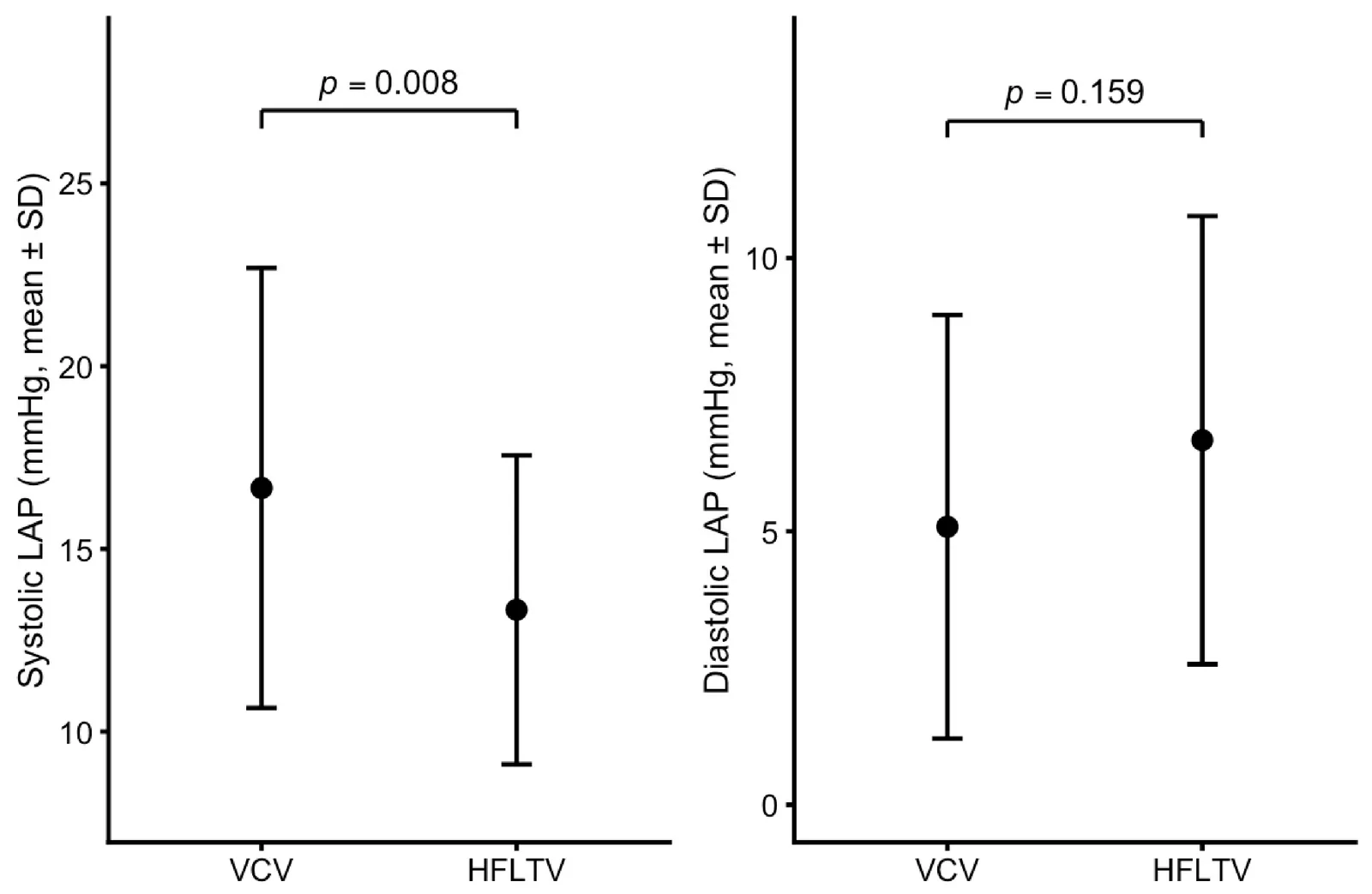
Comparison of systolic and diastolic left atrial pressure (LAP) during conventional volume-controlled ventilation (VCV) and higher-frequency low tidal ventilation (HFLTV). Data are presented as mean ± standard deviation (SD). Displayed *p*-values are derived from two-sided paired *t*-tests comparing HFLTV with VCV.

Systolic LAP was significantly lower during HFLTV compared to the measurement obtained during VCV (Table 4). Diastolic LAP showed no statistically significant difference between ventilation strategies. In the FDR sensitivity analysis, the difference in systolic LAP remained statistically significant after Benjamini–Hochberg adjustment. In the exploratory post hoc analysis, estimated mean LAP, derived as (systolic LAP + 2 × diastolic LAP)/3, was virtually unchanged between VCV and HFLTV (VCV 8.94 ± 4.29 vs. HFLTV 8.89 ± 3.85 mmHg; Mean Difference (MD) −0.06 mmHg, 95% confidence interval (CI) [−2.19; 2.08], *p* = 0.955). In contrast, the systolic-to-diastolic LAP amplitude was lower during HFLTV (VCV 11.58 ± 4.06 vs. HFLTV 6.67 ± 3.26; MD −4.92 mmHg, 95% CI [−6.72; −3.11], *p* < 0.001). Systolic and diastolic left atrial pressure values during conventional VCV and HFLTV, as well as results of paired two-sided *t*-testing and FDR-adjusted *p*-values are shown in Figure 2 and Table 4.

The patient-level mean values of heart rate, mean arterial pressure, and cardiac index obtained during HFLTV were higher than the corresponding measurements obtained during the initial VCV period, which remained statistically significant in the FDR sensitivity analysis (Table 4).

**Figure 3:**
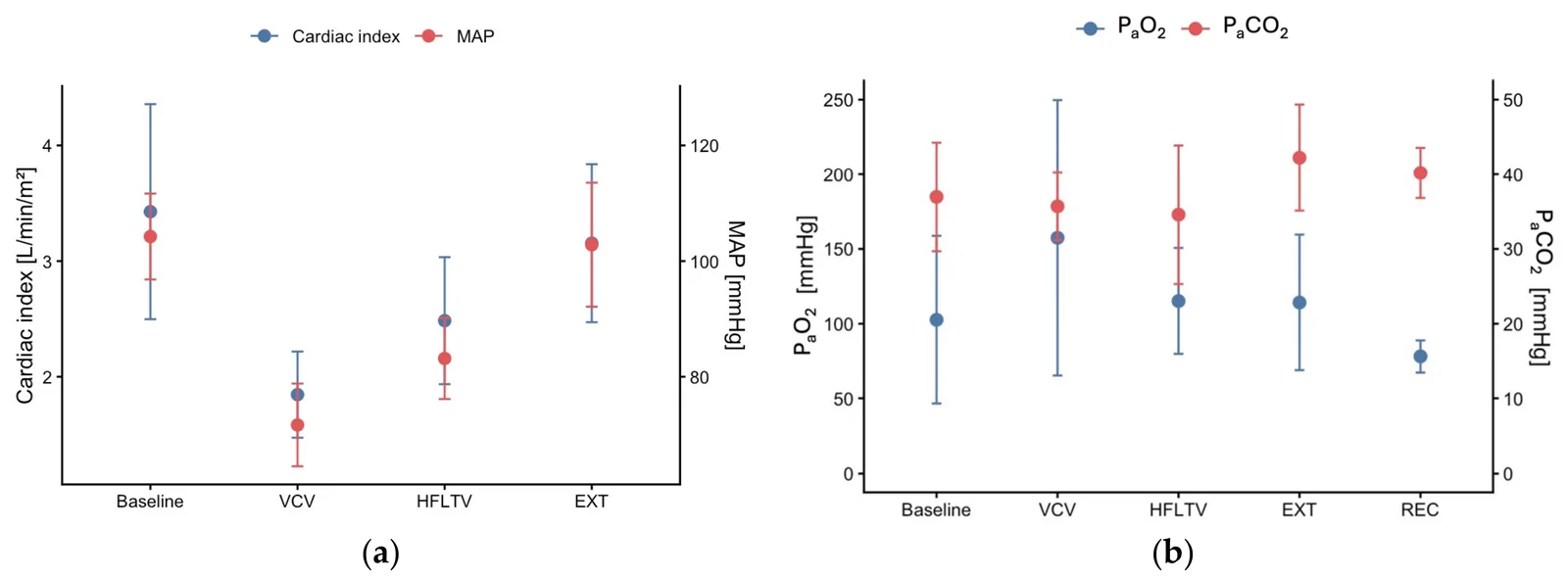
Chronological course of hemodynamic and respiratory parameters at baseline, during conventional volume-controlled ventilation (VCV) after induction, intubation, and recruitment, during higher-frequency low tidal ventilation (HFLTV), after extubation (EXT), and 1 h after admission to the recovery room (REC). HFLTV values represent the patient-level mean of repeated measurements obtained 5 min after initiation of HFLTV and subsequently at 30 min intervals. Data are presented as mean ± standard deviation (SD). For mean arterial pressure, P_a_O_2_, and P_a_CO_2_, data were available for all 12 patients at each displayed time point. For cardiac index, data were available for 11 patients at baseline, during VCV, during HFLTV, and after extubation (EXT); cardiac index was not available at the REC time point. (**a**) Cardiac index and mean arterial pressure. (**b**) Partial arterial pressures of oxygen (P_a_O_2_) and carbon dioxide (P_a_CO_2_). In line with the ventilation protocol, FiO_2_ was set to 0.40 during VCV and 0.50 during HFLTV. FiO_2_ was not protocol-defined for the baseline, EXT, and REC time points.

For arterial blood gases, the raw patient-level mean P_a_O_2_ during HFLTV was significantly lower than the corresponding VCV measurement (Table 4). Because FiO_2_ varied between measurement conditions, oxygenation was also assessed using the P/F ratio. This ratio was 351.3 ± 147.7 mmHg during VCV and 302.0 ± 93.3 mmHg during HFLTV. The P/F ratio was numerically lower during HFLTV, although the paired difference did not reach statistical significance (Table 4). P_a_CO_2_ did not differ significantly between ventilation conditions (Table 4). In the FDR sensitivity analysis, the difference in raw P_a_O_2_ did not remain statistically significant after adjustment. Hemodynamic and respiratory parameters during VCV and HFLTV are summarized in Table 4, Figure 3a,b and Appendix A, Table A1.

In the sensitivity analysis comparing the initial VCV measurement with the single measurement obtained 5 min after initiation of HFLTV, the directions of the differences were similar to those of the primary comparisons, but none reached statistical significance. The detailed results of this sensitivity analysis are displayed in Appendix A, Table A2.

**Figure A1:**
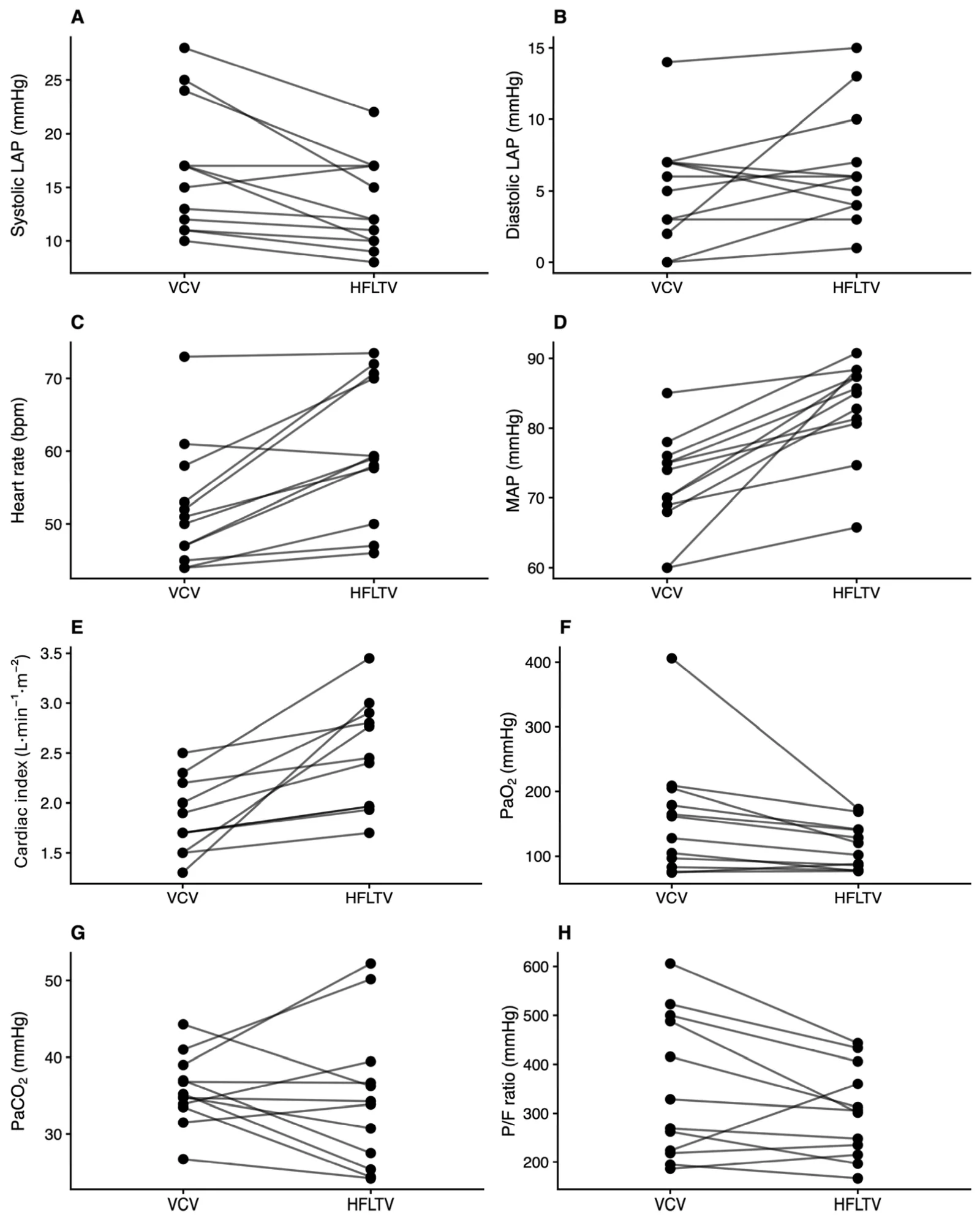
Individual patient-level paired observations during conventional volume-controlled ventilation (VCV) and higher-frequency low tidal ventilation (HFLTV). Paired observations are shown for (**A**) systolic left atrial pressure (LAP), (**B**) diastolic LAP, (**C**) heart rate, (**D**) mean arterial pressure (MAP), (**E**) cardiac index, (**F**) P_a_O_2_, (**G**) P_a_CO_2_, and (**H**) P_a_O_2_/FiO_2_ (P/F) ratio. Lines connect measurements from the same patient. For heart rate, MAP, cardiac index, P_a_O_2_, P_a_CO_2_, and the P/F ratio, the HFLTV value represents the patient-level mean of measurements obtained during HFLTV; for LAP, single paired measurements were used.

Assessment of the within-patient differences using the Shapiro–Wilk test indicated departures from normality for cardiac index (*p* = 0.011) and P_a_O_2_ (*p* < 0.001), but not for the remaining paired comparisons. Individual patient-level paired observations for all physiological outcomes are shown in Appendix A, Figure A1. In the non-parametric sensitivity analysis, the Wilcoxon signed-rank test yielded results consistent with the direction and overall interpretation of the paired *t*-test analyses. The Wilcoxon *p*-values were 0.014 for systolic LAP, 0.166 for diastolic LAP, 0.004 for heart rate, 0.003 for MAP, 0.004 for cardiac index, 0.009 for P_a_O_2_, 0.505 for P_a_CO_2_, and 0.078 for the P/F ratio.

Vasoactive support varied across the sequential ventilation and procedural periods and is detailed in Appendix A, Table A3. Norepinephrine was administered in 1 of 12 patients (8.3%) during the initial VCV measurement, compared with 3 of 12 patients (25.0%) at HFLTV initiation and 4 of 12 patients (33.3%) after 30 min of HFLTV, with a maximum documented infusion rate of 0.09 µg/kg/min. Additionally, intermittent cafedrine/theodrenaline administration was reported in 7 of 12 patients (58.3%), with a maximum cumulative dose of 120 mg cafedrine and 6 mg theodrenaline. No patient required rescue ventilation, interruption of HFLTV, recruitment maneuvers for desaturation, or treatment of arrhythmia during HFLTV.

## 4. Discussion

This single-center subgroup proof-of-concept analysis investigated physiological responses to a sequential higher-frequency low tidal volume ventilation protocol in twelve patients undergoing radiofrequency atrial fibrillation ablation. In this selected cohort, the HFLTV protocol was applied to all 12 patients throughout the procedural setting and was associated with differences in selected hemodynamic and respiratory parameters compared with conventional volume-controlled ventilation.

Regarding left atrial pressure, systolic LAP was lower during HFLTV, whereas diastolic LAP was numerically higher but did not differ significantly between ventilation conditions. Importantly, the post hoc estimated mean LAP was similar between ventilation periods, while the systolic-to-diastolic LAP amplitude was substantially reduced. However, this approximation was derived from systolic and diastolic LAP rather than directly measured from the atrial pressure waveform and should therefore be interpreted cautiously. Taken together, these findings indicate that the observed difference in systolic LAP occurred without a corresponding reduction in estimated mean left atrial filling pressure and was accompanied by a change in the pattern of recorded atrial pressures. Several factors may explain this observation. HFLTV represented a bundled ventilation protocol with simultaneous changes in respiratory rate, tidal volume, PEEP, FiO_2_, and I:E ratio. In particular, the reduction in PEEP from 5 to 8 cmH_2_O during VCV to 0 cmH_2_O during HFLTV may have altered intrathoracic pressure, venous return, and cardiac loading conditions. The magnitude of the expected pressure shift associated with the difference in PEEP may be of the same order as the observed systolic LAP difference; however, because intrathoracic pressure transmission and transmural LAP were not measured, the contribution of PEEP cannot be quantified from the present data. However, a simple uniform pressure shift would be expected to affect systolic and diastolic pressures in the same direction, whereas these changed in opposite directions in the present analysis. This suggests that the difference in PEEP alone may not fully account for the observed pattern. Other factors related to the fixed procedural sequence, including changes in anesthesia, ablation status, fluid balance, cardiac rhythm, atrial compliance, vasoactive support, or measurement characteristics, may also have contributed. Because transmural LAP and separate a- and v-wave amplitudes were not recorded, the underlying change in atrial pressure-wave morphology cannot be further characterized. Accordingly, the findings should be interpreted as a change in the recorded atrial pressure pattern between the sequential ventilation protocols rather than as evidence of reduced left atrial filling pressure or an isolated effect of higher respiratory frequency or lower tidal volume.

In addition to changes in LAP, HFLTV was associated with higher heart rate, mean arterial pressure, and cardiac index compared with VCV. These observations indicate measurable hemodynamic differences between ventilation conditions in this procedural setting. However, because several ventilatory parameters were modified simultaneously, these differences cannot be attributed to any individual component of the HFLTV protocol. Moreover, the fixed-order sequential design introduces potential time- and carryover-related confounding, as hemodynamic measurements were obtained at different procedural stages. Concurrent changes in anesthesiologic or procedural conditions may therefore have contributed to the observed differences. Importantly, the higher MAP and cardiac index observed during HFLTV cannot be separated from contemporaneous changes in vasoactive support, including the increased use of norepinephrine, and other procedural conditions and should therefore not be interpreted as isolated hemodynamic effects of the ventilation protocol. Vasoactive medication was administered during HFLTV in a subset of patients, with norepinephrine used in 4 of 12 patients and intermittent cafedrine/theodrenaline in 7 of 12 patients. Detailed time-resolved data on fluid administration and cardiac rhythm at each measurement time point were not available for this subgroup analysis. Consequently, the present design cannot distinguish differences associated with the ventilation protocol from concomitant anesthetic, hemodynamic, or procedural influences. Whether the observed changes represent clinically relevant differences or transient physiological adaptations to changing procedural conditions requires further investigation. The present exploratory analysis does not allow conclusions regarding causality or clinical benefit.

Regarding respiratory parameters, the interpretation of the observed difference in raw P_a_O_2_ requires caution because FiO_2_ varied between ventilation conditions and the nominally significant unadjusted comparison did not remain statistically significant after FDR adjustment. Therefore, the P/F ratio using the FiO_2_ recorded at each corresponding measurement was additionally assessed. The P/F ratio was numerically lower during HFLTV, although the difference did not reach statistical significance, with a wide confidence interval indicating considerable uncertainty in this small cohort. Importantly, FiO_2_ normalization does not account for the concurrent difference in PEEP, which was 5–8 cmH_2_O during VCV and 0 cmH_2_O during HFLTV. The absence of PEEP during HFLTV may have affected alveolar recruitment and ventilation–perfusion matching and thereby contributed to the observed oxygenation differences. Consequently, the present data do not allow the oxygenation findings to be attributed specifically to respiratory frequency or tidal volume. P_a_CO_2_ did not differ significantly between ventilation conditions. The numerically greater P_a_CO_2_–EtCO_2_ (end-tidal carbon dioxide) gradient during HFLTV may indicate altered dead space ventilation or ventilation–perfusion matching; however, the present data do not allow attribution to a specific mechanism. These findings may be considered in the context of previous reports suggesting that higher-frequency very low tidal volume ventilation can maintain short-term gas exchange during atrial fibrillation ablation. Ramírez-Paesano et al. recently demonstrated reduced respiration-related catheter displacement with high-frequency very low tidal volume ventilation with controlled peak inspiratory pressure limitation in a subgroup pilot study of 52 patients [10]. Their findings, including preserved oxygenation and controlled permissive hypercapnia, supported the physiological feasibility of this approach while emphasizing that these results should be interpreted as evidence of short-term tolerance rather than clinical superiority [10]. The present analysis extends these observations by providing additional invasive assessment of left atrial pressure and concurrent hemodynamic measurements.

Previous investigations of high-frequency ventilation during interventional procedures have mainly focused on procedural advantages, such as reduction in respiratory motion and improved catheter stability [12,13]. However, comprehensive evaluation of cardiovascular and respiratory responses remains limited. The present analysis contributes additional physiological data by combining invasive cardiac pressure measurements with respiratory and hemodynamic parameters during a standardized sequential ventilation protocol.

This study has several limitations. First, the sample size of twelve patients was small and the analysis was restricted to a preselected subgroup of patients enrolled in the Ablate-by-LAWT study [9]. Therefore, findings should be considered exploratory and hypothesis-generating. Second, a central limitation is the fixed-order sequential design. The ventilation conditions were neither randomized nor counterbalanced, and paired measurements were obtained at different procedural stages. Consequently, the comparisons are inherently subject to time- and sequence-related confounding and potential carryover effects. Changes in anesthesia depth, procedural and ablation status, fluid balance, autonomic tone, cardiac rhythm, vasoactive medication, and other time-dependent factors may have contributed to the observed differences. In addition, the primary comparisons of HR, MAP, CI, and blood gas parameters were analytically asymmetric, as a single early VCV measurement was compared with the patient-level mean of repeated measurements obtained throughout the HFLTV period. In a sensitivity analysis using single measurements from initial VCV and 5 min after initiation of HFLTV, the directions of the observed differences were similar, but their magnitudes differed and none reached statistical significance. This further limits interpretation of the primary paired comparisons and suggests that the results may depend partly on the selection and aggregation of measurement time points. Detailed time-resolved data on fluid administration, catheter irrigation, and diuresis were not available, precluding assessment of their potential contribution to the observed hemodynamic and left atrial pressure differences. The present design cannot separate these effects from those associated with the ventilation protocols. Accordingly, the paired within-patient comparisons should be interpreted as exploratory physiological observations rather than estimates of an isolated effect of HFLTV. Third, the mechanisms underlying the observed differences in LAP and hemodynamic parameters cannot be determined from the available measurements. Fourth, HFLTV was implemented as a bundled ventilation protocol involving simultaneous changes in respiratory rate, tidal volume, PEEP, FiO_2_, and the I:E ratio. Consequently, the observed differences cannot be attributed specifically to higher respiratory frequency or lower tidal volume. In particular, the protocol-defined external PEEP of 0 cmH_2_O during HFLTV may have contributed to the observed hemodynamic differences through changes in intrathoracic pressure and cardiac loading conditions. Furthermore, total PEEP was not measured using end-expiratory occlusion maneuvers. Thus, the presence and magnitude of intrinsic PEEP or dynamic hyperinflation during HFLTV cannot be determined. Plateau pressure was not systematically recorded, precluding calculation of driving pressure and limiting assessment of respiratory mechanical stress. Finally, no prespecified physiological tolerance or safety criteria were defined, and the present exploratory subgroup analysis was not designed or powered to establish the safety or physiological tolerance of HFLTV. Although no rescue ventilation, interruption of HFLTV, recruitment for desaturation, or treatment of arrhythmia was required in this small cohort, these observations should not be interpreted as evidence of safety. Accordingly, the reported respiratory, acid–base, and hemodynamic parameters should be considered exploratory short-term physiological observations.

Despite these limitations, the present analysis adds invasive left atrial pressure measurements to the limited physiological data available for higher-frequency low tidal volume ventilation during atrial fibrillation ablation. Future randomized and counterbalanced studies incorporating direct atrial pressure-waveform analysis, standardized assessment of total PEEP, and detailed control of time-dependent procedural factors are needed to clarify the physiological effects and potential clinical relevance of this ventilation strategy.

## 5. Conclusions

In this exploratory single-center subgroup analysis, a sequential higher-frequency low tidal volume ventilation protocol was applied in 12 selected patients undergoing radiofrequency atrial fibrillation ablation. Differences in left atrial pressure, hemodynamic parameters, and oxygenation were observed between the sequential HFLTV and VCV periods. Given the fixed-order design and the simultaneous modification of multiple ventilatory parameters, these observations cannot be attributed specifically to the HFLTV protocol or any of its individual components and should be considered hypothesis-generating. Randomized, counterbalanced studies with comprehensive respiratory and hemodynamic monitoring, including direct left atrial pressure-waveform assessment, are needed to clarify the physiological and clinical relevance of these findings.

## Figures and Tables

**Table 1:** Comparison of ventilatory parameters of conventional volume-controlled ventilation (VCV) and higher-frequency low tidal ventilation (HFLTV) (FiO2: Fraction of inspired oxygen, I:E ratio: Inspiration-to-expiration ratio, PEEP: Positive end-expiratory pressure, RR: Respiratory rate, TV: Tidal volume).

Ventilatory Parameter	VCV	HFLTV
FiO_2_ [%]	40	50
TV [mL/kg]	6–8	3.5–4
RR [breaths/minute]	12–15	45–55
I:E ratio	1:2 ^1^	1:1.5
PEEP [cmH_2_O]	5–8 ^1^	0

^1^ During VCV, the I:E ratio and PEEP were individually titrated to optimize ventilation.

**Table 2:** Baseline characteristics and procedural data (ASA: American Society of Anesthesiologists, BMI: Body mass index, CAD: Coronary artery disease, EHRA: European Heart Rhythm Association, HFLTV: Higher-frequency, low tidal ventilation, IQR: interquartile range, MI: Myocardial infarction, SD: Standard deviation, TIA: Transient ischemic attack).

	Overall	
Variable	12	
Female sex (*n*, %)	5	(41.67)
Age [years] (mean, SD)	61.83	(7.42)
BMI (mean, SD)	24.93	(4.33)
Height [cm] (mean, SD)	177.0	(8.32)
Weight [kg] (mean, SD)	77.75	(11.58)
LVEF [%] (median, IQR)	57	(53–60)
Smoking status (*n*, %)		
Never	6	(50.0)
Former	3	(25.0)
Current	3	(25.0)
CAD (*n*, %)		
No CAD	7	(58.3)
1-vessel disease	3	(25.0)
2-vessel disease	2	(16.7)
Arterial hypertension (*n*, %)	8	(66.7)
Previous MI (*n*, %)	2	(16.7)
Previous stroke/TIA (*n*, %)	1	(8.3)
Beta-blocker therapy (*n*, %)	10	(83.3)
Antiarrhythmic therapy (*n*, %)	2	(16.7)
Preprocedural EHRA class (*n*, %)		
EHRA I	0	(0.0)
EHRA II	4	(33.3)
EHRA III	5	(41.7)
EHRA IV	3	(25.0)
Preprocedural CHA_2_DS_2_-VASc-Score (median, IQR)	2.0	(1.5–2.0)
Preprocedural ASA-Score (*n*, %)		
ASA I	0	(0.0)
ASA II	0	(0.0)
ASA III	10	(83.3)
ASA IV	2	(16.7)
Baseline SpO_2_ [%] (mean, SD)	97.4	(2.4)
Duration of anesthesia [minutes] (mean, SD)	134.67	(27.57)
Duration of intervention [minutes] (mean, SD)	97.00	(16.13)
Duration of HFLTV [minutes] (mean, SD)	85.67	(12.15)

**Table 3:** Measured ventilatory and respiratory parameters during volume-controlled ventilation (VCV) and the higher-frequency low tidal volume protocol (HFLTV) (RR: Respiratory rate, TV: Tidal volume, MV: Minute volume, P_insp_: Inspiratory pressure, EtCO_2_: End-tidal carbon dioxide, P_a_CO_2_: Partial arterial pressure of carbon dioxide, SpO_2_: Peripheral oxygen saturation).

Parameter	VCV	HFLTV
RR [breaths/min]	11.4 ± 1.4	49.0 ± 5.2
TV [mL]	593.2 ± 92.1	261.6 ± 34.1
MV [L/min]	7.1 ± 1.5	13.6 ± 3.6
P_insp_ [cmH_2_O]	15.7 ± 4.6	15.8 ± 6.3
Compliance [mL/cmH_2_O] ^1^	83.1 ± 17.6	61.8 ± 20.4
EtCO_2_ [mmHg]	27.6 ± 3.9	23.0 ± 4.3
P_a_CO_2_–EtCO_2_ [mmHg]	8.1 ± 4.7	10.5 ± 5.0
SpO_2_ [%]	99.0 ± 1.3	98.2 ± 1.5
pH	7.45 ± 0.04	7.45 ± 0.07

Data are presented as mean ± SD. For VCV, values represent the measurements obtained during the initial VCV period after induction, intubation, and recruitment. For HFLTV, repeated measurements obtained during HFLTV were averaged within each patient before calculation of the group-level mean and SD. ^1^ Compliance represents ventilator-reported compliance rather than independently measured respiratory system compliance.

**Table 4:** Paired comparison of hemodynamic and respiratory parameters during VCV and HFLTV (sLAP: Systolic left atrial pressure; dLAP: Diastolic left atrial pressure; HR: Heart rate; MAP: Mean arterial pressure; CI: Cardiac index; P_a_O_2_: Partial pressure of arterial oxygen; P_a_CO_2_: Partial pressure of arterial carbon dioxide; P/F: P_a_O_2_/FiO_2_ ratio or Horovitz index).

Parameter	*n*	VCV	HFLTV	MD (95% CI)	*p*	FDR-adj. *p*
sLAP [mmHg]	12	16.67 ± 6.02	13.33 ± 4.23	−3.33 [−5.61; −1.06]	0.008	0.016
dLAP [mmHg]	12	5.08 ± 3.87	6.67 ± 4.10	1.58 [−0.72; 3.89]	0.159	0.182
meanLAP [mmHg]	12	8.94 ± 4.29	8.89 ± 3.85	−0.06 [−2.19; 2.08]	0.955	- ^1^
s-dLAP [mmHg]	12	11.58 ± 4.06	6.67 ± 3.26	−4.92 [−6.72; −3.11]	<0.001	- ^1^
HR [bpm]	12	52.1 ± 8.5	60.2 ± 9.6	8.1 [3.8; 12.4]	0.002	0.006
MAP [mmHg]	12	71.7 ± 7.2	83.2 ± 7.0	11.5 [7.1; 15.9]	<0.001	0.004
CI [L/min/m^2^]	11	1.85 ± 0.37	2.48 ± 0.55	0.64 [0.29; 0.99]	0.002	0.006
P_a_O_2_ [mmHg]	12	157.4 ± 92.2	115.2 ± 35.5	−42.3 [−83.5; −1.1]	0.045	0.072
P_a_CO_2_[mmHg]	12	35.7 ± 4.5	34.6 ± 9.3	−1.1 [−6.0; 3.7]	0.623	0.623
P/F [mmHg]	12	351.3 ± 147.7	302.0 ± 93.3	−49.3 [−105.2; 6.7]	0.079	0.105

Data are presented as mean ± SD. MD represents the paired mean difference (HFLTV − VCV) with a 95% confidence interval. Unadjusted *p*-values were obtained using two-sided paired *t*-tests. FDR-adjusted *p*-values were calculated using the Benjamini–Hochberg procedure to account for multiple comparisons. *n* indicates the number of complete paired observations included in the respective analysis. ^1^ Post hoc exploratory analysis; not included in the FDR adjustment of the original physiological outcomes. Estimated mean LAP was calculated as (systolic LAP + 2 × diastolic LAP)/3, and LAP amplitude as systolic minus diastolic LAP.

## Data Availability

The raw data supporting the conclusions of this article will be made available by the authors on request due to restrictions.

## Abbreviations

The following abbreviations are used in this manuscript:

AF	Atrial Fibrillation
ASA	American Society of Anesthesiologists
BMI	Body Mass Index
CAD	Coronary Artery Disease
CI	Cardiac Index
CI	Confidence Interval
EHRA	European Heart Rhythm Association
EtCO_2_	End-tidal Carbon Dioxide
EXT	Extubation
FDR	False Discovery Rate
FiO_2_	Fraction of Inspired Oxygen
HFvLTV-cPP	High-Frequency, Very Low Tidal Volume Ventilation with Controlled Peak Pressure
HFLTV	Higher-Frequency, Low Tidal Ventilation
HR	Heart Rate
I:E	Inspiration to Expiration Ratio
IQR	Interquartile Range
LAP	Left Atrial Pressure
LAWT	Left Atrial Wall Thickness
LVEF	Left Ventricular Ejection Fraction
MAP	Mean Arterial Pressure
MD	Mean Difference
MI	Myocardial Infarction
MV	Minute Volume
P_a_CO_2_	Partial Arterial Pressure of Carbon Dioxide
P_a_O_2_	Partial Arterial Pressure of Oxygen
PEEP	Positive End-Expiratory Pressure
P/F	P_a_O_2_/FiO_2_ Ratio
REC	Recovery Room
RR	Respiratory Rate
SD	Standard Deviation
SpO_2_	Peripheral Oxygen Saturation
TIA	Transient Ischemic Attack
TV	Tidal Volume
VCV	Volume-Controlled Ventilation

## Appendix A

**Table A1:** Hemodynamic and respiratory parameters after extubation (EXT) and in the recovery room (REC) (MAP: Mean arterial pressure; CI: Cardiac index; P_a_O_2_: Partial pressure of arterial oxygen; p_a_CO_2_: Partial pressure of arterial carbon dioxide).

Parameter	After Extubation ^1^	In the Recovery Room ^1^
MAP [mmHg]	102.8 ± 10.7	100.4 ± 12.4
CI [L/min/m^2^]	3.15 ± 0.68	N.A.
P_a_O_2_ [mmHg]	114.2 ± 45.4	78.0 ± 10.8
P_a_CO_2_ [mmHg]	42.2 ± 7.1	40.2 ± 3.4

^1^ Data are presented descriptively as mean ± SD.

**Table A2:** Sensitivity analysis comparing the initial VCV measurement with the 5-min HFLTV measurement (HR: Heart rate; MAP: Mean arterial pressure; CI: Cardiac index; P_a_O_2_: Partial pressure of arterial oxygen; P_a_CO_2_: Partial pressure of arterial carbon dioxide; P/F: P_a_O_2_/FiO_2_ ratio or Horovitz index).

Parameter	VCV	HFLTV	MD (95% CI)	*p*
HR [bpm]	52.1 ± 8.5	56.0 ± 14.1	3.9 [−3.8; 11.6]	0.285
MAP [mmHg]	71.7 ± 7.2	77.3 ± 10.4	5.6 [−1.1; 12.2]	0.092
CI [L/min/m^2^]	1.85 ± 0.37	1.97 ± 0.36	0.13 [−0.17; 0.43]	0.365
P_a_O_2_ [mmHg]	157.4 ± 92.2	120.7 ± 41.4	−36.7 [−78.9; 5.5]	0.082
P_a_CO_2_ [mmHg]	35.7 ± 4.5	33.3 ± 7.1	−2.4 [−5.6; 0.8]	0.132
P/F [mmHg]	351.3 ± 147.7	332.0 ± 105.0	−19.3 [−89.4; 50.9]	0.558

Data are presented as mean ± SD. MD represents the paired mean difference (HFLTV − VCV) with a 95% confidence interval. Unadjusted *p*-values were obtained using two-sided paired *t*-tests. VCV represents the initial measurement obtained after induction, intubation, and recruitment; HFLTV represents the single measurement obtained 5 min after initiation of HFLTV.

**Table A3:** Vasoactive support (NE: norepinephrine [µg/kg/min]; C/T: cafedrine/theodrenaline [mL, 1 mL containing 20 mg/1 mg]) across the sequential ventilation and procedural periods.

Time Point	Available, *n*	NE, *n* (%)	NE Rate ^1^	C/T, *n* (%)	C/T, Dose ^1^
VCV	12	1 (8.3)	0.010 (0.010–0.010)	4 (33.3)	2 (2–3)
Initiation of HFLTV	12	3 (25.0)	0.020 (0.015–0.035)	5 (41.7)	2 (2–2)
HFLTV + 30 min	12	4 (33.3)	0.015 (0.010–0.038)	3 (25.0)	2 (2–2)
HFLTV + 60 min	12	2 (16.7)	0.055 (0.043–0.068)	1 (8.3)	2 (2–2)
HFLTV + 90 min	3	1 (33.3)	0.040 (0.040–0.040)	0 (0.0)	-
After HFLTV	12	1 (8.3)	0.020 (0.020–0.020)	1 (8.3)	2 (2–2)
After Extubation	12	0 (0.0)	-	1 (8.3)	2 (2–2)

^1^ Doses are presented as the median (IQR) among patients receiving the respective medication.
